# Supplementary material for: A Bioassay-Guided Fractionation of Rosemary Leaf Extract Identifies Carnosol as a Major Hypertrophy Inducer in Human Skeletal Muscle Cells
Source: Nutrients. 2021 Nov 23;13(12):4190. doi: 10.3390/nu13124190 (PMC8706380; doi:10.3390/nu13124190)
Supplement: Supplementary file 1 [file nutrients-13-04190-s001.zip › nutrients-1421754-supplementary.pdf]

A bioassay-guided fractionation of rosemary leaf extract identifies carnosol as a major hypertrophy inducer in human skeletal muscle cells.

Sylvie Morel[1], Gérald Hugon[2], Manon Vitou[1], Marie Védère[2], Françoise Fons[1], Sylvie Rapior[1], Nathalie Saint [2,a] and Gilles Carnac[2,a,b]

1- Laboratoire de Botanique, Phytochimie et Mycologie, Faculté de Pharmacie, CEFE, Univ Montpellier, CNRS, EPHE, IRD, Montpellier, France.

2- PhyMedExp, Université de Montpellier, CNRS, INSERM, Montpellier, France.

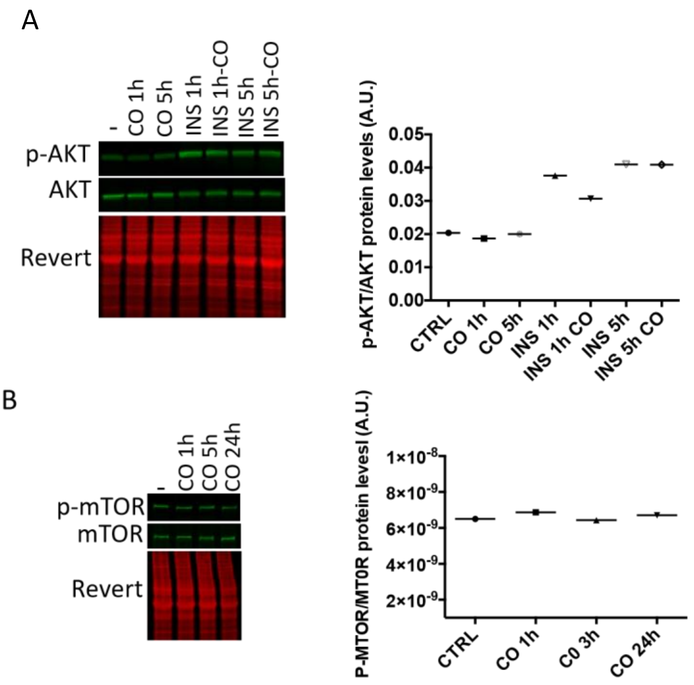

Figure S1: The Akt/mTOR pathway is not activated in response to carnosol incubation.

At confluence, myoblasts (M19) were induced to differentiate for 3 days, and then, carnosol (CO, 1 µg/mL), insulin (INS, 100nM) or carnosol + insulin were added to the culture for the indicated times. A) On the left: myotube protein extracts were analyzed by western blotting with anti-phosphorylated AKT (pAKT) and -total AKT antibodies. Revert® total protein stain was used as internal loading control. On the right: quantification of the western blotting data; A.U., arbitrary units (i.e. the ratio of pAKT/AKT relative to the Revert® stain level). B) On the left: myotube protein extracts (treated as before) were analyzed by western blotting with antiphosphorylated mTOR (p-mTOR) and -total mTOR protein antibodies. Revert® total protein stain was used as internal loading control. On the right: quantification of the western blotting data; A.U., arbitrary units (i.e. the ratio of p-mTOR/mTOR relative to Revert® stain level; M19, 19-year-old man donor.

A bioassay-guided fractionation of rosemary leaf extract identifies carnosol as a major hypertrophy inducer in human skeletal muscle cells.

Sylvie Morel[1], Gérald Hugon[2], Manon Vitou[1], Marie Védère[2], Françoise Fons[1], Sylvie Rapior[1], Nathalie Saint [2,a] and Gilles Carnac[2,a,b]

1- Laboratoire de Botanique, Phytochimie et Mycologie, Faculté de Pharmacie, CEFE, Univ Montpellier, CNRS, EPHE, IRD, Montpellier, France.

2- PhyMedExp, Université de Montpellier, CNRS, INSERM, Montpellier, France.

A

B

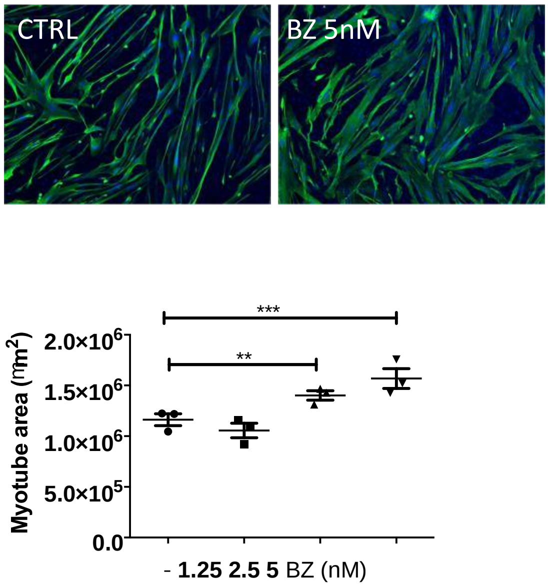

Figure S2: The proteasome inhibitor bortezomib increases myotube cell size

At confluence, myoblasts (M19) were induced to differentiate for three days, and then, incubated (BZ) or not (CTRL) with increasing concentrations of bortezomib for 48 hours. Myotubes were fixed and analyzed by indirect immunofluorescence. (A) Representative images showing troponin T staining (green). DNA was stained with DAPI (blue). (B) Quantification of troponin T immunofluorescence images with the Fiji software; **p <0.01, ***p <0.001. Data are the mean ± SD of three biological replicates. M19, 19-year-old man donor.
